# Development and validation of a SEER-based prognostic nomogram for patients with bone metastatic prostate cancer

**DOI:** 10.1097/MD.0000000000017197

**Published:** 2019-09-27

**Authors:** Guangdong Hou, Yu Zheng, Di Wei, Xi’an Li, Fuli Wang, Jingyang Tian, Geng Zhang, Fei Yan, Zheng Zhu, Ping Meng, Jiarui Yuan, Ming Gao, Zhibin Li, Bin Zhang, Zibao Xing, Jianlin Yuan

**Affiliations:** a Department of Urology, Xijing Hospital, the Air Force Medical University, Xi’an; b Department of Otorhinolaryngology, Hainan Hospital of Chinese PLA General Hospital, Sanya, P.R. China; c St. George's University School of Medicine, Grenada, West Indies; d Assisted Reproduction Center, Northwest Women's and Children's Hospital, Xi’an, P.R. China.

**Keywords:** bone metastases, nomogram, prognosis, prostate cancer, SEER

## Abstract

Controversies exist between the previous two prognostic nomograms for patients with bone metastatic prostate cancer (PCa), and a nomogram applied to western patients has yet to be established. Thus, we aimed to build a reliable and generic nomogram to individualize prognosis.

The independent prognostic factors were identified in a retrospective study of 1556 patients with bone metastatic PCa registered in the Surveillance, Epidemiology and End Results (SEER) database. Besides, the prognostic nomogram was developed using R software according to the result of multivariable Cox regression analysis. Then, the discriminative ability of the nomogram was assessed by analyses of receiver operating characteristic curves (ROC curves). We also performed 1-, 2-, and 3-year calibrations of the nomogram by comparing the predicted survival to the observed survival. Furthermore, the model was externally validated using the data of 711 patients diagnosed at different times enrolled in the SEER database.

Age ≥70 years, Gleason score ≥8, PSA value of 201 to 900 ng/ml, stage T4, stage N1, with liver metastases, and Asian/Pacific ethnicity were identified as independent prognostic factors. In the primary cohort, 1-, 2-, and 3-year area under the ROC curve (AUC) of the nomogram for predicting cancer-specific survival (CSS) were 0.71, 0.70, and 0.70, respectively. Besides 1-, 2-, and 3-year AUC were 0.70, 0.68, and 0.69, respectively, in the external validation cohort. Moreover, calibration curves presented perfect agreements between the nomogram-predicted and actual 1-, 2-, and 3-year CSS rate in both the primary and external validation cohorts. In other words, our nomogram has great predictive accuracy and reliability in predicting 1-, 2-, and 3-year CSS for patients with bone metastatic prostate cancer.

This study established and validated a prognostic nomogram applied to not only Asian patients but western patients with bone metastatic PCa, which will be useful for patients’ counseling and clinical trial designing.

## 1 Introduction

Prostate cancer (PCa) is the second most common malignancy and the fifth leading cancer-related cause of death in men worldwide, with nearly 1.3 million newly diagnosed cases and about 359,000 deaths in 2018.^[1,2]^ Metastatic disease is often present at the first diagnosis in the clinic, among which bone metastases are the most common sites in PCa, accounting for about 14% of all cases at initial diagnosis.^[3]^ With the occurrence of bone metastases, patients experience bone pain, pathological fracture, and other symptoms that negatively affect their quality of life.^[4,5]^ Worse still, the 5-year survival rate of patients with bone metastases was significantly lower than that of those without bone metastases (3% vs 56%).^[6]^

In the past two decades, androgen axis therapies have made remarkable advances; however, the overall survival (OS) and cancer-specific survival (CSS) of patients with metastatic PCa have not improved.^[7]^ At present, the independent prognostic factors for bone metastatic PCa remain controversial and there are significant differences in OS between studies.^[8,9]^ It should also be noted that prostate-specific antigen (PSA) values were >20 ng/ml in over 90% patients with bone metastatic PCa, which means almost all patients enter the D’Amico high-risk stage when bone metastases are present.^[3]^ Therefore, nomograms based on the equation derived from the regression coefficients of each variable always integrates many prognostic factors may better predict their survival.^[10]^ Unfortunately, noticeable differences and even wholly contrary opinions exist regarding the contribution of some factors in the previous two prognostic models (namely the Indonesian and Japanese models) for patients with bone metastatic PCa, which may lead to confusion among urologists and patients.^[8,11]^ Furthermore, in view of geographic and ethnic differences, these two models can only be applied within Indonesia or Japan. For these reasons, there is an urgent need to obtain accurate information on factors correlated with survival and to develop a prognostic model that can be applied to not only Asian patients but also western patients.

The present study represents one of the most extensive series to investigate prognostic factors for patients with bone metastatic PCa. Moreover, this is the first nomogram that can be applied to multi-racial patients with bone metastatic PCa.

## 2 Materials and methods

### 2.1 Study design

The SEER database supported by the National Cancer Institute (NCI) is a population-based cancer registry covering approximately 30% of the United States population.^[12]^ After obtaining approval from the NCI, we searched a total of 316,724 cases of PCa registered between 2010 and 2015. We included only cases with a histological subtype of adenocarcinoma (8140/3, according to the third edition of International Classification of Diseases for Oncology [ICD-O-3]); PSA values of 20 to 900 ng/ml; and non-Hispanic white (NHW), non-Hispanic black (NHB), Hispanic (HIS), and Asian/Pacific Islander (A/PI) ethnicities. Patients were excluded from our cohort if PCa was not the first tumor for patients, if the cases were diagnosed without or with unknown bone metastases, if the cases were diagnosed through death certificate or at autopsy only, if the cases were missing clinical or demographic data (age, ethnicity, marital status, PSA value, Gleason score, T stage, N stage, the presence of liver or lung metastases, and survival status), and if the cases were T0 stage. Furthermore, cases diagnosed after January 1, 2015, were excluded to ensure that all cases had undergone observation of survival status for more than 1 year when the last follow-up was conducted on December 2015. Finally, 2267 cases were eligible for our study. The 1156 patients who were diagnosed between January and August of each year from 2010 to 2014 were included in the primary cohort, while the 711 patients diagnosed between September and December of each year from 2010 to 2014 constituted the validation cohort. The present study conformed to the 1964 Helsinki Declaration and its later amendments, and was approved by the research ethics board of Xijing Hospital of the Air Force Medical University.

### 2.2 Covariates and follow-up information

The covariates extracted from the SEER database included the patients’ demographic characteristics (namely age, ethnicity, and marital status), PSA value, Gleason score, T stage, N stage, and the presence or absence of liver/lung metastases. As we were aiming to establish a pre-treatment nomogram, we did not consider treatment variables. In addition, we only included cases with PSA value between 20 and 900 ng/ml because the SEER database does not provide specific values over 980 and patients with PSA levels <20 ng/ml only account for about 3% of all patients with bone metastatic PCa.^[3]^ Finally, continuous variables (age and PSA value) were transformed into categorical variables according to the median number in the analysis.

The starting point of follow-up was the date of diagnosis with bones metastatic PCa. The endpoint was cancer-specific death or the last follow-up in December 2015. The data of patients who were lost to follow-up, died due to other causes, or who survived to the last follow-up were censored.

### 2.3 Statistical analysis

Statistical analyses to identify the prognostic factors were performed in SPSS 22.0 (IBM Corp., Armonk, NY, USA). The Kaplan–Meier method was used to estimate the CSS and OS. The significance of differences in CSS was assessed by log-rank tests. Variables that achieved significance at *P* < .1 in univariate Cox regression were entered into multivariate Cox proportional hazard models for further analysis.

The nomogram was developed according to the results of multivariate Cox analysis and using the rms package in R version 3.5.1 (http://www.r-project.org/). The predictive accuracy of the nomogram was measured by ROC curve analysis. Besides, 1-, 2-, and 3-year calibrations of the nomogram were performed by comparing the predicted survival to the observed survival. Bootstraps with 1000 resamples were used for these evaluations. The data were extracted using SEER∗Stat Software version 8.3.5. Differences with *P* ≤ .05 (two-sided) were considered statistically significant.

## 3 Results

### 3.1 Characteristics of patients and survival outcomes

The demographic and clinicopathologic characteristics of the primary and external validation cohorts are presented in Table 1.

**Table 1:**
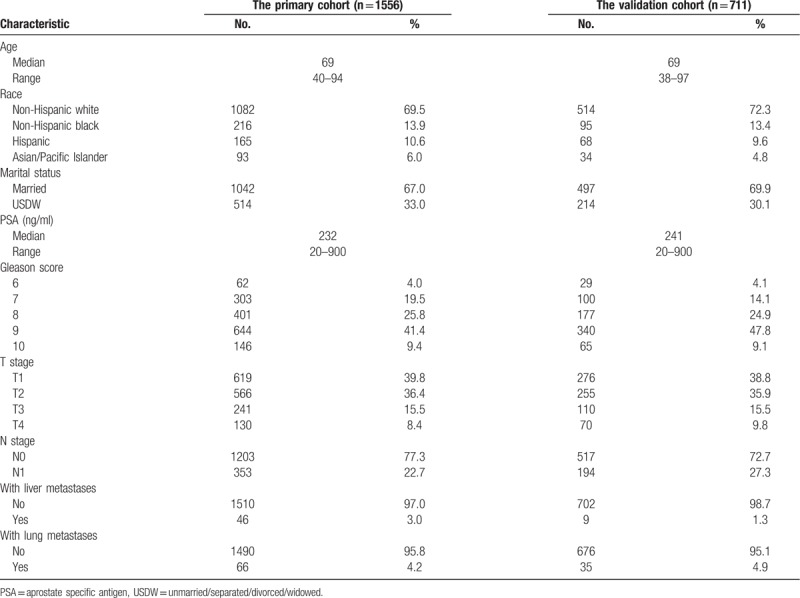
Characteristics of patients with bone metastatic prostate cancer.

In the primary cohort, after a median follow-up of 25 months (interquartile range: 16–42 months), 46.7% (726/1556) of patients died, and 39.2% (610/1556) died due to PCa up to the last follow-up conducted in December 2015. The median CSS was 51 months (95% CI, 46.063–55.937), with 1-, 2-, and 3-year CSS rates of 88.1%, 71.6%, and 60.5%, respectively. The median OS was 41 months (95% CI, 37.093–44.907), with 1-, 2-, and 3-year OS rates of 85.6%, 67.5%, and 55.0%, respectively.

In the validation cohort, after a median follow-up of 24 months (interquartile range: 13–38 months), 41.4% (294/711) of patients died, and 33.3% (237/711) died due to PCa. The median CSS was 54 months (95% CI, 45.798–62.202), with 1-, 2-, and 3-year CSS rates of 87.6%, 73.4% and 60.4%, respectively. In addition, the median OS was 40 months (95% CI, 34.463–45.537) and the 1-, 2-, and 3-year OS rates were 84.8%, 68.2%, and 54.2%, respectively.

### 3.2 Independent prognostic factors for CSS in the primary cohort

Through univariable analysis and subsequent multivariable Cox analysis, patient age ≥70 (*P* < .001, hazard ratio [HR] = 1.436, 95% CI, 1.219–1.691); PSA of 201-900 ng/ml (*P* = .011, HR = 1.236, 95% CI, 1.050–1.455); Gleason scores of 8 (*P* = .009, HR = 2.209, 95% CI, 1.217–4.010), 9 (*P* < .001, HR = 3.359, 95% CI, 1.872–6.028), and 10 (*P* < .001, HR = 4.495, 95% CI, 2.435–8.295); T4 stage (*P* = .002, HR = 1.546, 95% CI, 1.174–2.035); N1 stage (*P* = .018, HR = 1.255, 95% CI, 1.039–1.516); and combined with liver metastases (*P* < .001, HR = 3.642, 95% CI, 2.548–5.204) were independent risk predictors for CSS in patients with bone metastases PCa. In addition, A/PI ethnicity (*P* = .001, HR = 0.474, 95% CI, 0.305–0.737) was an independent protective factor for CSS compared to NHW as the reference. As shown in Table 2.

**Table 2:**
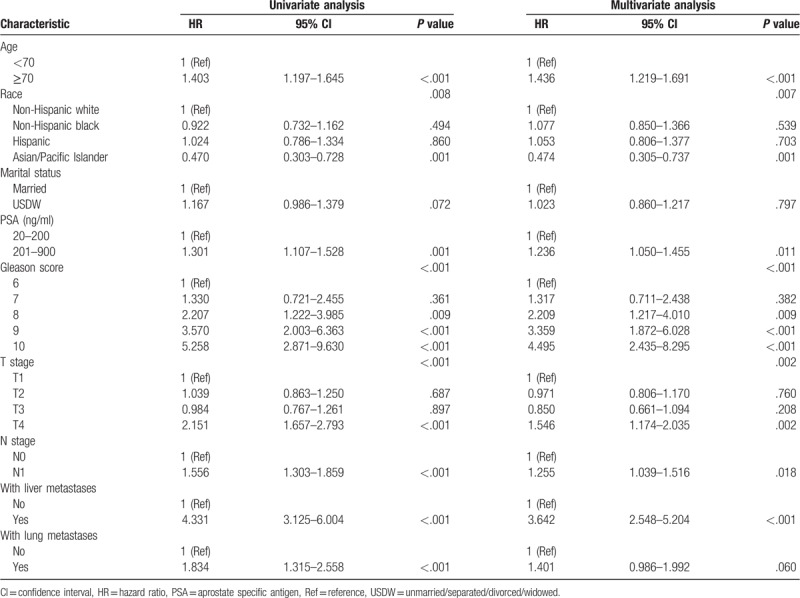
Univariate and Multivariate Cox analysis of the patients with bone metastatic prostate cancer in the primary cohort.

### 3.3 Development of a prognostic nomogram for CSS

**Figure 1:**
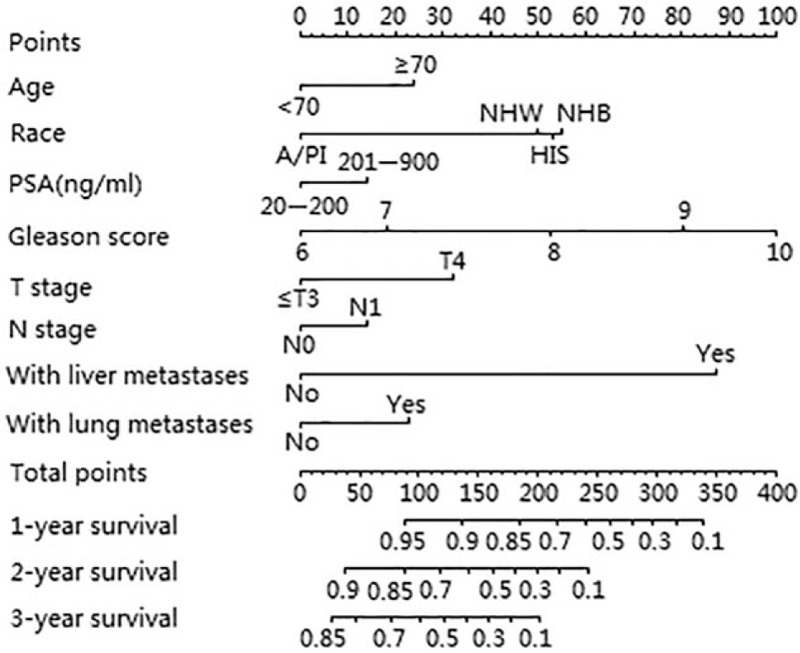
Nomogram predicting 1-, 2-, and 3-year cancer-specific survival (CSS) for patients with bone metastatic PCa.

The prognostic nomogram was developed by integrating all independent factors for CSS in the primary cohort, besides, we also include the variable of lung metastases, whose *P*-value was very close to .05. The length of the line corresponding to each variable in the nomogram represents the contribution of predictors to survival outcomes. The nomogram showed that Gleason score made the most significant contribution to the survival outcome, closely followed by the presence/absence of liver metastases. In addition, ethnicity had a moderate impact on prognosis. Moreover, T stage, age, the presence of lung metastases, N stage, and PSA value had relatively weak contributions to the survival outcome (Fig. 1).

Each subtype of the variables contributing to the nomogram corresponded to a point on the “Points” scale. We can calculate the total points for a particular patient with bone metastatic PCa by summing each score corresponding to the subtype for each variable. A straight line can then be drawn from the location of these total points on the “Total points” scale to provide the probability of 1-, 2-, and 3-year CSS for the individual patient.

### 3.4 Validation and calibration of the nomogram for CSS

**Figure 2:**
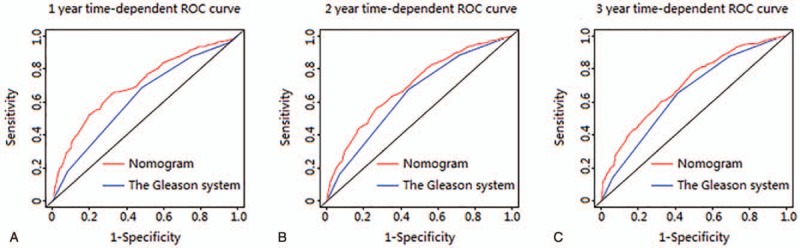
ROC curves of the Nomogram and the Gleason system for predicting 1-, 2-, and 3-year cancer-specific survival (CSS) in the primary cohort.

The discriminative ability of the nomogram was measured using values of 1-, 2-, and 3-year time-dependent AUC (AUC value equal to 0.5 indicates that the nomogram has no predictive effect, and AUC value equal to 1 indicates that the nomogram can completely distinguish patients with different survival rates. The higher value between 0.5 and 1, the better discriminative ability of the nomogram), and its superiority was further verified by comparing with the Gleason system, which made the most significant contribution to survival in our nomogram. In the primary cohort, the nomogram showed strengths in the discriminative ability compared with the Gleason system (1-year AUC: 0.71 vs 0.62, 2-year AUC: 0.70 vs 0.64, 3-year AUC: 0.70 vs 0.65, Fig. 2). Besides, in the external validation cohort, values of 1-, 2-, and 3-year AUC were 0.70, 0.68, and 0.69, respectively.

**Figure 3:**
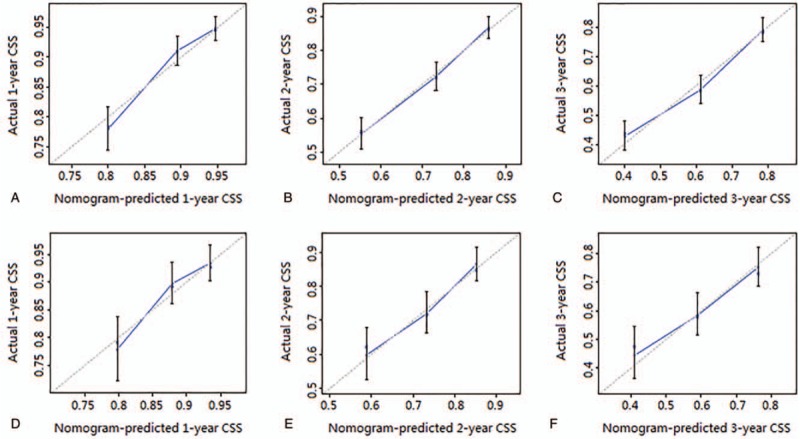
(A–C) The calibration curves of nomogram for predicting 1-, 2-, and 3-year CSS in the primary cohort. (D–F) The calibration curves of nomogram for predicting 1-, 2-, and 3-year CSS in the external validation cohort. Nomogram-predicted CSS is plotted on the x-axis; actual CSS is plotted on the y-axis. The imaginary line indicates a perfect calibration model in which the predicted probabilities are identical to the actual survival outcomes.

Moreover, calibration curves were all very close to perfect curves (curves corresponding to perfect situations in which nomogram-predicted CSS rate is exactly the same as the actual CSS rate) at 1, 2, and 3 years after the first diagnosis in both the primary cohort and the external validation cohort. In other words, perfect agreements were achieved between the nomogram-predicted and actual 1-, 2-, and 3-year CSS rates, which guaranteed the reliability of the nomogram, as indicated by the calibration plots (Fig. 3).

## 4 Discussion

PCa is the most commonly diagnosed malignancy among men in more than half of the countries worldwide and is the leading cause of cancer-related death among men in 46 countries.^[1]^ Moreover, the occurrence of bone metastases can threaten patients’ quality of life and survival. However, independent prognostic factors for bone metastatic PCa remain controversial. For this reason, one aim of our study was to identify the independent prognostic predictors for patients with bone metastatic PCa.

A nomogram is a graphical representation of a multivariable prognostic model that integrates many prognostic factors and can be used to evaluate individual probabilities of survival at a certain time accurately. Nomograms have been built for several cancers and have been shown to be more accurate than traditional tools in predicting prognosis.^[13–16]^ Nomograms also have the highest predictive accuracy and a superior discriminating ability for predicting survival in patients with PCa compared to those of other prediction tools.^[17]^ Over the last 15 years, several prognostic nomograms for patients with PCa have been established; however, only two models (the Indonesian and Japanese models) focused exclusively on patients presenting with bone metastases.^[18,19]^ Unfortunately, significant differences regarding the contribution of age, and opposite opinions related to the contribution of PSA exist between the two models. Moreover, both models can only be used for Asian populations, in view of geographic and ethnic differences, a nomogram applied to westerners has yet to be established. For these reasons, the main aim of our study was to develop a generic model on the basis of large samples, which could be applied to not merely Asian patients but also western patients with bone metastatic PCa.

Through univariable analysis and subsequent multivariable analysis, we identified Gleason score ≥8 and T4 stage as independent risk factors for CSS, concordant with the findings of the previous Indonesian and Japanese studies. Age also played a crucial role in our model, similar to that in the Japanese model. However, age was not included in the Indonesian nomogram because it was not an independent predictor for OS. This difference may be caused by the insufficient sample size of the Indonesian cohort.

More interestingly, with the increase in PSA valve, the prognosis worsened in the Indonesian nomogram, completely opposite to the finding that patients with a higher PSA value have a more favorable prognosis in the Japanese study. The completely contrary opinions between the previous two models for patients with bone metastatic PCa are confusing when predicting patient survival. Fortunately, based on large samples, we confirmed that high PSA is associated with inferior prognosis; however, the predictive contribution of PSA in the bone metastases PCa-specific nomogram is very weak.

In addition, we identified N1 stage and the presence of liver metastases as independent risk predictors for CSS, consistent with another study also using data from the SEER database.^[20]^ However, to our knowledge, our study is the first one to include N stage and liver and lung metastasis status into models for patients with bone metastasis PCa. In our nomogram, the presence of liver metastases, which is probably because of lymphovascular invasion, made a decisive contribution to an inferior prognosis. Therefore, we strongly recommend that patients with bone metastatic PCa undergo immediate upper abdominal CT examination to determine whether liver metastasis is also present to better predict prognosis and adopt a reasonable treatment regimen. In the present study, only 10 PCa patients had bone plus brain metastases after excluding cases with incomplete clinical or demographic data; thus, as this sample did not meet statistical requirements, we did not include information on brain metastases as a variable in our analyses.

Ethnic differences in survival have been reported in patients with PCa.^[21,22]^ The results of our study confirmed that NHW had an approximately two-fold increased risk of death compared to that of A/PI and that there were almost no survival differences among NHW, NHB, and HIS. Petrovics et al reported different spectrums of genomic alterations between African-American and Caucasian-American patients with PCa; thus, the differences in survival between A/PI and other ethnicities may also be due to different spectrums of genes.^[23]^ Because of the ethnic differences in survival, there are considerable limitations in the use of the Indonesian and Japanese models. With broader applicability, our model is the first nomogram for western patients with bone metastatic PCa and can be used in almost all patients with bone metastatic PCa worldwide by incorporating ethnicity as a variable in the nomogram, which may be the greatest strength of the present study.

The present study was based on data from the SEER database, with a large sample size and sufficient data. However, our study has several limitations. First, as we aimed to develop a pre-treatment nomogram, we did not include treatment variables, by adding which might increase the predictive accuracy of the nomogram. In addition, we only included patients who had presented with bone metastases at first diagnosis and did not include those who developed bone metastases at later times, who were not enrolled in the SEER database. Furthermore, although they may also be strong independent prognostic factors, we did not analyze alkaline phosphatase and hemoglobin levels, as these variables are not available in the SEER database. Moreover, patients with missing data with respect to each of the variables were excluded from our cohort, which may have increased the bias. Despite these limitations, the performance of our model are ensured, as evidenced by the agreements between the nomogram-predicted and actual 1-, 2-, and 3-year CSS, with 1-, 2-, and 3-year AUC of 0.71, 0.70, and 0.70, respectively.

## 5 Conclusion

Age ≥70 years, Gleason score ≥8, PSA value of 201 to 900 ng/ml, T4 stage, N1 stage, and the presence of liver metastases were identified as independent risk factors and that A/PI ethnicity was an independent protective factor for survival in patients with bone metastatic PCa. Furthermore, we established a reliable and generic prognostic nomogram for application to not only Asian but Western patients with bone metastatic PCa, by which 1-, 2-, and 3-year CSS can be predicted individually and accurately. However, further validation using external data is required to generalize the applicability of our model in clinical practice.

## Author contributions

Study designed: Jianlin Yuan. Searching of data: Jiarui Yuan. Statistical analysis: Guangdong Hou and Zheng Zhu. Analysis and interpretation of data: Yu Zheng, Fuli Wang, Geng Zhang, Fei Yan and Zhibin Li. Drafting of the manuscript: Guangdong Hou. Figs designed: Xi’an Li and Jingyang Tian. Searching of literature: Di Wei, Bin Zhang, and Zibao Xing. Obtained funding: Ping Meng and Ming Gao.

**Data curation:** Jiarui Yuan.

**Formal analysis:** Yu Zheng, Fuli Wang, Geng Zhang, Fei Yan.

**Funding acquisition:** Ping Meng, Ming Gao.

**Methodology:** Jianlin Yuan.

**Resources:** Di Wei, Zhibin Li, Bin Zhang, Zibao Xing.

**Software:** Xi’an Li, Jingyang Tian, Zheng Zhu.

**Supervision:** Fuli Wang.

**Writing – original draft:** Guangdong Hou.
